# Antimicrobial Activity of Synthetic Enterocins A, B, P, SEK4, and L50, Alone and in Combinations, against *Clostridium perfringens*

**DOI:** 10.3390/ijms25031597

**Published:** 2024-01-27

**Authors:** Sara García-Vela, Louis-David Guay, Md Ramim Tanver Rahman, Eric Biron, Carmen Torres, Ismail Fliss

**Affiliations:** 1 Area of Biochemistry and Molecular Biology, OneHealth-UR Research Group, University of La Rioja, 26006 Logrono, La Rioja, Spain; sara.garcia-vela1@ulaval.ca; 2 Department of Food Science, Laval University, Quebec, QC G1V 0A6, Canada; 3 Institute of Nutrition and Functional Foods, Laval University, Quebec, QC G1V 0A6, Canada; louis-david.guay.2@ulaval.ca (L.-D.G.); mrrah@ulaval.ca (M.R.T.R.); eric.biron@pha.ulaval.ca (E.B.); 4 Faculty of Pharmacy, Laval University, Quebec, QC G1V 0A6, Canada; 5 Laboratory of Medicinal Chemistry, CHU de Québec Research Center, Quebec, QC G1V 4G2, Canada

**Keywords:** enterocins, antimicrobial activity, *Clostridium perfringens*, chemical synthesis, synergy

## Abstract

Multidrug-resistant *Clostridium perfringens* infections are a major threat to the poultry industry. Effective alternatives to antibiotics are urgently needed to prevent these infections and limit the spread of multidrug-resistant bacteria. The aim of the study was to produce by chemical synthesis a set of enterocins of different subgroups of class II bacteriocins and to compare their spectrum of inhibitory activity, either alone or in combination, against a panel of twenty *C. perfringens* isolates. Enterocins A, P, SEK4 (class IIa bacteriocins), B (unsubgrouped class II bacteriocin), and L50 (class IId leaderless bacteriocin) were produced by microwave-assisted solid-phase peptide synthesis. Their antimicrobial activity was determined by agar well diffusion and microtitration methods against twenty *C. perfringens* isolates and against other pathogens. The FIC_INDEX_ of different combinations of the selected enterocins was calculated in order to identify combinations with synergistic effects. The results showed that synthetic analogs of L50A and L50B were the most active against *C. perfringens*. These peptides also showed the broadest spectrum of activity when tested against other non-clostridial indicator strains, including *Listeria monocytogenes*, methicillin-resistant *Staphylococcus aureus*, *Streptococcus suis*, *Streptococcus pyogenes*, *Enterococcus cecorum*, *Enterococcus faecalis*, as well as Gram-negative bacteria (*Campylobacter coli* and *Pseudomonas aeruginosa*), among others. The selected synthetic enterocins were combined on the basis of their different mechanisms of action, and all combinations tested showed synergy or partial synergy against *C. perfringens*. In conclusion, because of their high activity against *C. perfringens* and other pathogens, the use of synthetic enterocins alone or as a consortium can be a good alternative to the use of antibiotics in the poultry sector.

## 1. Introduction

Antimicrobial resistance is a serious public health problem that compromises the treatment of infections in both humans and animals. This problem is mainly linked to the unnecessary prescription and/or misuse of antibiotics. In addition to the clinical use of antibiotics in humans, they are also used in veterinary medicine and animal husbandry to treat and prevent infections, and even in agriculture to preserve crops, even if at low levels. Antibiotics have also been used extensively as growth promoters in food-producing animals, but although this practice has been banned in Europe since 2006 and in other countries, it is still allowed in some others [1,2]. In countries where antibiotic growth promoters are no longer used, infections such as poultry-associated necrotic enteritis (NE) induced by *Clostridium perfringens* have increased. Some of these *Clostridium* strains are multidrug-resistant [3], which suggests that this may also be the case for other relevant pathogens [4]. Necrotic enteritis caused by *C. perfringens* is one of the most common poultry diseases and causes huge economic losses in poultry farming [5]. Effective alternatives to antibiotics are needed to prevent the spread of multidrug-resistant bacteria and the development of emergent infections in the poultry industry.

Among the most promising alternatives, bacteriocins show very attractive properties [6]. Bacteriocins are ribosomally synthesized peptides that have antimicrobial activity against bacteria closely related to the producing strain. Enterococci are ubiquitous microorganisms that can be found everywhere: in water, plants, soil, food, and the gastrointestinal tract of humans and animals [7]. They have been used traditionally as starters in food fermentation, as protective cultures in food biopreservation or as probiotics, as they produce bacteriocins called enterocins [8,9]. In recent years, the direct use of enterococci as a starter or as a probiotic has generated an important debate due to the presence of virulence and antibiotic resistance genes and the high risk associated with genetic transfer mechanisms [10]. Thus, the use of their antimicrobial products instead of isolates could be a promising alternative to the use of antibiotics.

Enterocins are short cationic peptides (20–60 amino acids) with hydrophobic sections, which are highly stable to heat and over a wide range of pH [11]. In general, they have activity against phylogenetic species close to the producing bacteria, but some of them exhibit a broad-spectrum of activity, including Gram-positive microorganisms such as *Listeria monocytogenes*, *Bacillus cereus*, *Staphylococcus* spp., and *Clostridium* spp.; Gram-negative microorganisms such as *Pseudomonas aeruginosa*, *Escherichia coli*, or *Vibrio cholera*; and even against fungi and viruses [12,13]. Enterocins have several advantages when used as an alternative to antibiotics. Namely, their narrow spectrum of action causes less destabilization of the microbiota and their potency makes them very effective. Moreover(in addition), their sensitivity to proteases ensures biosafety and they can be modified by bioengineering, which makes them easy to handle.

In general, bacteriocins are most commonly produced by bacterial fermentation using the producing strains. However, the low production yields combined with difficulties associated with their purification severely limit their potential for large-scale use. Chemical synthesis has been proposed as an alternative to producing several bacteriocins such as pediocin PA-1 [14] and bactofencin A [15]. The main advantage of this approach is the increase in the speed at which large quantities of pure bacteriocins can be produced. In addition, the significant reduction in the cost of peptide synthesis reagents and building blocks has made the chemical synthesis of bacteriocins more attractive and competitive [16].

For this study, enterocins of different classes, namely enterocin A (EntA), enterocin B (EntB), enterocin P (EntP), enterocin SEK4 (EntSEK4), and enterocin L50 (L50) were selected and produced by chemical synthesis. These class II bacteriocins are unmodified, low molecular weight (<10 kDa), and thermostable bacteriocins, which do not involve the use of non-proteogenic amino acids. Therefore, no special enzymes other than signal peptides or transporters are required to complete the maturation and activation of such bacteriocins [17].

Enterocin A, enterocin P, and enterocin SEK4 are class IIa bacteriocins (pediocin-like bacteriocins) containing the consensus YGNGV sequence and a disulfide bond formed by two cysteines in the N-terminal section, both being signatures of this class. As class IIa bacteriocins, they bind to the mannose phosphotransferase (ManPTS) receptor in order to form a pore in the target gram-positive membrane [18,19]. 

Enterocin L50 does not have a consensus sequence and is composed of the two peptides L50A and L50B that are synthesized without a leader peptide being classified as leaderless bacteriocins, included in class IId [19,20]. Most leaderless bacteriocins do not require binding to a receptor for their killing activity [17,20]. Enterocins L50A and L50B seem to be receptor-independent, membrane-directed bacteriocins [19], and they possess an N-terminal formylated methionine. Finally, enterocin B is a non-subgrouped class II linear bacteriocin, and its mechanism of action remains unknown [10].

In general, pediocin-like class IIa bacteriocins act by forming pores in the membrane of Gram-positive bacteria via their interaction with the mannose phosphotransferase system (Man-PTS), as is the case for enterocin A, P, and SEK4 [21,22,23]. The Man-PTS system consists of 4 subunits: IIA, IIB, IIC, and IID. The phosphotransfer subunits IIA and IIB are not required for the interaction, but the subunits IIC and IID are involved in the mechanism of action of these bacteriocins. However, there is controversy as to whether IIC or IID are involved in the creation of bacteriocin pores, or whether their role is simply to aid membrane penetration and pore assembly. Sensitivity to bacteriocins is correlated with the expression level of the receptor/target protein, but also mutations of the target (subunit IID) can attenuate this sensitivity [24].

The enterocins used in this study have been reported to have antimicrobial activity against several pathogenic bacteria. Enterocin A was first identified in 1996 and is produced by several *Enterococcus faecium* strains [25]. Enterocin A shows activity against *Enterococcus* spp., *Lactobacillus* spp., *Pediococcus* spp., and *L. monocytogenes* [25]. Enterocin A is usually co-produced with enterocin B, which was initially produced by *E. faecium* T136 isolated from Spanish fermented sausages [26]. Enterocin B shows antimicrobial activity against Gram-positive bacteria such as *L. monocytogenes*, *Propionibacterium* spp., *Clostridium sporogenes*, and *Clostridium tyrobutyricum*. When enterocin A and enterocin B are co-produced, they form a heterodimer, and studies have demonstrated its potential antibacterial and anti-biofilm activities against *Staphylococcus aureus*, *Acinetobacter baumannii*, *L. monocytogenes*, and *E. coli* [27]. Enterocin P is produced by *E. faecium* P13 isolated from Spanish fermented sausages. The spectrum of activity of enterocin P includes *Lactobacillus* spp., *Pediococcus* spp., *Propinobacterium* spp., and *Enterococcus* spp. and the pathogens *L. monocytogenes*, *S. aureus*, *C. perfringens*, and *Clostridium botulinum* [28]. Enterocin SEK4 was first identified in *Enterococcus faecalis* K-4 isolated from grass silage growing at 43–45 °C and has antimicrobial activity against *Enterococcus* spp., *Bacillus subtilis*, *Clostridium. beijerinckii*, and *L. monocytogenes* [29]. Enterocin L50 was first detected in an *E. faecium* L50 strain isolated from Spanish fermented sausage [30]. It consists of two peptides, L50A and L50B, which synergistically promote their antimicrobial activity. Enterocin L50 A/B has a broad spectrum of antimicrobial activity, including *Enterococcus* spp., *Lactobacillus* spp., *Lactococcus lactis*, *Pediococcus pentosaceus*, *L. monocytogenes*, *S. aureus*, *B. cereus*, *C. botulinum*, *Streptococcus* spp., and *C. perfringens* [31]. 

Although there is a great amount of information available on the inhibitory activity of several enterocins, this information has been obtained using disparate isolates of various origins and using different in vitro methods. To our knowledge, no study has compared the inhibition spectrum of enterocins with different mechanisms of action against a panel of bacteria using the same method at the same time. 

The aim of this study is to produce by chemical synthesis enterocins belonging to different subclasses inside the bacteriocins of class II and to compare their spectrum of inhibitory activity, either alone or in combination against a large panel of *C. perfringens* and other relevant bacteria.

## 2. Results

### 2.1. Production of the Enterocins

Linear enterocins A, B, P, SEK4, L50A, and L50B were successfully produced by microwave-assisted solid-phase peptide synthesis. After release from the solid support and deprotection of the side chains, each enterocin was purified by preparative HPLC and characterized by mass spectrometry (MS) (Supplementary Figure S1). The synthesized enterocins were obtained in purities greater than 95% in 3–10% overall yields. No disulfide bond formation was performed for enterocins A, B, P, and SEK4 during synthesis, and they have been used as is in the antimicrobial assays since the in situ formation of a disulfide bond has been recently demonstrated with the use of linear and cyclic pediocin PA-1 and bactofencin A showing the same activity [14,15]. Native enterocins L50A and L50B contain an N-terminal N-formylated methionine residue, but non-formylated analogs have been synthesized in this study and used in the antimicrobial assays.

### 2.2. Antimicrobial Activity of the Enterocins against C. perfringens Isolates

**Figure 1:**
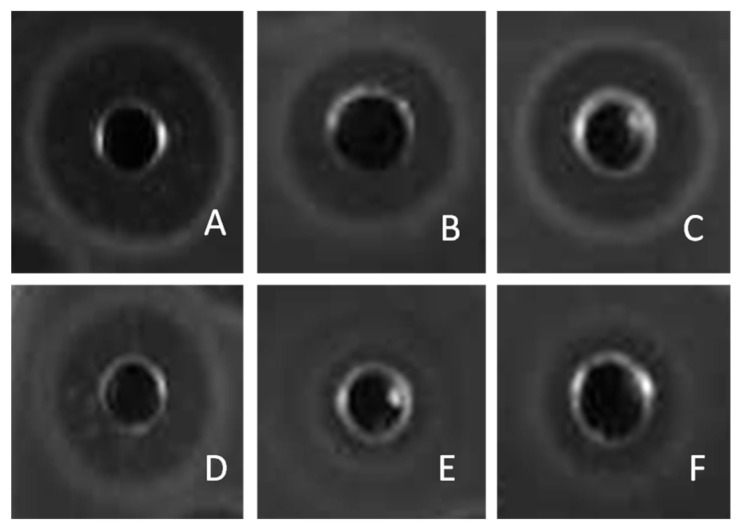
Inhibition halos obtained with enterocin A (**A**); enterocin P (**B**), enterocin L50A (**C**), enterocin L50B (**D**), enterocin SEK4 (**E**), and enterocin B (**F**) against *C. perfringens* MLG3111. The wells contained 80 µL of each enterocin at a concentration of 200 µg/mL in water.

The zones of inhibition obtained in the agar well diffusion assays with the enterocins against the collection of *C. perfringens* isolates are included in Supplementary Table S1. Figure 1 shows inhibition halos for the enterocins against the susceptible strain *C. perfringens* MLG3111. For these results, only clear inhibition halos were considered positive. Enterocin A, enterocin B, enterocin P, and the two peptides of enterocin L50 inhibited the growth of the entire *C. perfringens* collection, whereas enterocin SEK4 only showed inhibitory activity against five of the 20 *C. perfringens* isolates. The most active enterocin tested against *C. perfringens* was enterocin A, with an average diameter of the inhibition halos of 20.3 mm. 

The minimum inhibitory concentration (MIC) values of the different enterocins against the collection of *C. perfringens* isolates are shown in Table 1. Enterocin L50A and L50B showed the lowest MICs, being the most active, followed by enterocin B and enterocin A. Enterocin SEK4 and enterocin P showed very high MICs. 

### 2.3. Whole Genome Sequencing Analysis

Pairwise alignments of the product of the genes encoding the IID subunit of the Man-PTS (*manZ_1*, *manZ_2*, and *manZ_3*) revealed differences between the *C. perfringens* ATCC 13124 strain and the isolates from our collection (Supplementary Figures S2–S4). Regarding the product of *manZ_1*, similarities can be observed between the three *C. perfringens* isolates from the U. Laval collection, with *C. perfringens* MLG 7307 clearly being different from the other two of them. The alignments of the four isolates can be visualized in Supplementary Figure S2. The ATCC 13124 *man_Z1* product of *C. perfringens* presented a 31.80% similarity with *C. perfringens* MLG 2919. Among the many mutations observed, we can highlight the presence of two deletions: one deletion of 12 amino acids from positions p207 to p218; and another of 18 amino acids from position p232 to p249. About the similarities and differences in the *man_Z1* product of the *C. perfringens* isolates from the collection, *C. perfringens* MLG 0418 (one of the most susceptible to the class II enterocins) and MLG 2319 (one of the less susceptible) showed a 99.96% of identity, finding one substitution in p294, in which an Ala is replaced by a Val in MLG 2919. Comparing them with the *manZ_1* product of MLG 7307, which had the lowest MICs of the study, with MLG 0418 and MLG 2919, we detected identities of 93.40 and 93.73%, respectively. The MLG7307 *manZ_1* product presented 13 substitutions in comparison with the *manZ_1* product of MLG 0418 with the *manZ_1* product of MLG 0418 and 14 substitutions in the *manZ_1* product of MLG 2919.

Of the similarities of the product of *manZ_2*, only two of the isolates of the collection (MLG 0418 and MLG 2919) were identical (100% identity). They presented 30.29% identity with *C. perfringens* ATCC 13124 and 29.39% identity with the *manZ_2* products of the MLG 7307 isolate. The alignments can be visualized in Supplementary Figure S3.

For the *manZ_3* product, the MLG 7307 strain did not present that gene in its genome. Comparing the products of this gene of the other two isolates of the *C. perfringens* collection, an identity of 99.63% was detected between the ones of MLG 2919 and MLG 0418, with a substitution in Asn55Lys. The alignments can be visualized in Supplementary Figure S4.

### 2.4. Antimicrobial Activity of the Enterocins against Other Relevant Pathogens

**Figure 2:**
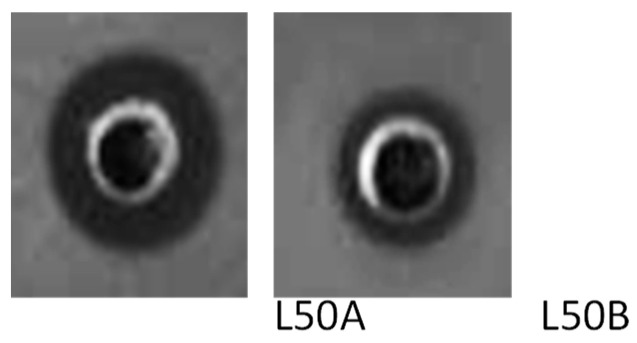
Inhibition halos of the two peptides of enterocin L50 (L50A, L50B), against the Gram-negative *P. aeruginosa* ATCC 27855. The wells contained 80 µL of each enterocin at a concentration of 200 µg/mL in water.

All synthesized enterocins showed strong activity against *L. monocytogenes*. Enterocins L50A and L50B showed the broadest spectrum of activity and were even active against the Gram-negative *P. aeruginosa* ATCC 27855 and *C. coli* ATCC 33559 (Figure 2). Inhibition diameters (in mm) from the enterocins against the other relevant bacteria used as indicators are shown in Supplementary Table S2. The MIC values obtained against these pathogens are represented in Table 2. While the results showed that *L. monocytogenes* was the most sensitive strain to the tested enterocins, enterocins L50A and L50B yielded the widest spectrum of activity, with L50A exhibiting the lowest MICs.

### 2.5. Synergistic Effects of Different Enterocin Combinations

To evaluate the synergistic, additive, and antagonistic effects, the FIC index was calculated using the following combinations of enterocins: L50A-L50B, EntA-EntB, EntA-L50A, EntA-L50B, EntB-L50A, EntB-L50B, EntP-L50A, and EntP-L50B. *C. perfringens* MLG 3111 was chosen as the indicator strain due to its high sensitivity to enterocins. The results are shown in Table 3. Four combinations resulted in synergistic effects and the other four resulted in partial synergy. Synergistic combinations were EntA-L50A, EntA-L50B, EntP-L50A, and EntP-L50B.

## 3. Discussion

Enterocins A, B, P, SEK4, L50A, and L50B were successfully obtained by microwave-assisted solid-phase peptide synthesis, highlighting the potential of chemical synthesis to produce long peptides (i.e., >40 AA) such as bacteriocins. Because the in situ formation of a disulfide bond in biological media has been recently demonstrated with linear and cyclic pediocin PA-1 and bactofencin A, showing the same activity [14,15], no disulfide bond formation was performed during the synthesis, and linear enterocins A, B, P, and SEK4 were used as is in the antimicrobial assays. In this study, N-terminal free enterocins L50A and L50B have been used instead of the native *N*-formylated form. There is controversy about the effect of formylation of the N-terminal methionine residue on the activity of these leaderless bactericions. In this sense, some authors indicate that N-terminal formylation increases antimicrobial activity [32], while others report that the N-terminal formyl group may not have a significant role in the bioactivity of leaderless bacteriocins as in the case of L50A and L50B [20]. Further studies are currently underway to optimize the synthesis steps and increase yields. Access to these enterocins by chemical synthesis allows modifications to be made to optimize their physicochemical and pharmacological properties as well as further studies for their use in the food and animal production industry. Another production method that could be considered as an alternative to fermentation is the use of recombinant microorganisms, as is the case with *Pichia pastoris*. This has been completed in several studies with enterocins such as enterocins HF, A, CRL35, P, and Hiracin JM79, among others [33,34,35].

All the enterocins produced showed antimicrobial activity against the complete *C. perfringens* collection, with the exception of enterocin SEK4, which showed antimicrobial activity against only five of these isolates, even though it has shown activity against clostridial isolates in other studies [28]. The MIC values of class IIa enterocin A and enterocin P were around 100 µg/mL, although they showed large inhibition halos in the agar well diffusion assay. The situation was different with enterocin B. Even when the halo was small, the MICs were lower than those of the class IIa enterocins. This could be attributed to the fact that enterocin B is a large peptide and may not diffuse properly through the agar pores. Both peptides of enterocin L50 showed promising results in terms of antimicrobial activity against the whole *C. perfringens* collection, with the L50A peptide being more active than the L50B peptide. The L50A peptide even showed MIC values very close to those produced by nisin, which is known to be very active [29]. The rest of the enterocins exhibited higher MIC values than nisin, although they were still active at low concentrations. Considering that the enterocins selected for this study have different modes of action, enterocins that bind to the manPTS receptor (class IIa) showed similar MICs against *C. perfringens* isolates. The same phenomenon occurred with enterocin L50, which exhibits a different mechanism of action, and nisin, which binds to the lipid II receptor [36,37]. Enterocin B, whose receptor has not yet been identified, showed MIC values similar to those of enterocins A and P. 

The products of the different genes encoding the subunit IID of the Man-PTS system showed different substitutions and deletions when compared with isolates of different susceptibility levels and with the reference strain *C. perfringens* ATCC 13124. Since many polymorphisms were detected, no conclusions regarding differences in the susceptibility of the strains could be obtained. Additionally, the subunit II+C could not be analyzed since its coding sequence was not detected in the analysis. However, as resistance to pediocin-like IIa bacteriocins is not only due to mutations in the Man-PTS system but also to overexpression of the genes [24], it cannot be concluded that these differences in the amino acid sequences of the subunit IID of the Man-PTS system are the only ones responsible for the differences in enterocin susceptibility. Further studies, such as qPCRs analyzing the level of expression of the genes, are required to complete the explanation. Likewise, the differences in susceptibility to the pediocin-like class IIa enterocins were not very remarkable.

In terms of the spectra of activity, all enterocins produced were active against *L. monocytogenes* ATCC 1911 with very low MICs. Pediocin-like antimicrobial peptides have previously been used to control foodborne pathogens such as *Listeria* [38]. This study highlights the fact that they can be used for this purpose. It also shows that they can be produced by chemical synthesis, which facilitates their purification and further uses [14]. In addition to *Listeria*, class IIa enterocins were also active against other relevant bacteria. These included *E. faecalis* ATCC 29212, *S. suis* C2058, and *S. pyogenes* ATCC 19615. Typically, bacteriocins act against closely related bacteria because they are produced to compete for the ecological niche [31]). That is the case for enterocin A and enterocin B, which are active against *E. faecalis* ATCC 29212. In this study, enterocin P and enterocin SEK4 were only active against *L. monocytogenes* ATCC 1911, representing a very narrow spectrum of activity, and this may be positive for applications targeting only this pathogen. In contrast, enterocin L50 showed a broader spectrum of activity, with both peptides also active against the tested Gram-negative bacterial strains, which is not common for bacteriocins produced by Gram-positive bacteria [30,38]. Both L50A and L50B peptides are promising antimicrobial peptides for further studies, not only because of their broader spectrum of action but also because of their high activity at low concentrations. Therefore, they can be effective not only against Gram-positive poultry pathogens such as *C. perfringens* and *E. cecorum*, which cause huge damage in the poultry sector [39,40,41], but also against Gram-negative pathogens such as *C. coli* and *P. aeruginosa*. In addition, L50A and L50B are active against *S. aureus* ATCC 6538 and also against the methicillin-resistant *S. aureus* C411. This underlines the idea that they can be used as an alternative to antibiotics in the case of multidrug-resistant bacteria.

The FIC_INDEX_ was calculated to evaluate the activity of different enterocin combinations. All combinations tested had partial or synergistic effects, supporting the idea that the combination of enterocins with different modes of action can be used to enhance antimicrobial activity. The combinations EntA-L50A, EntA-L50B, EntP-L50A, and EntP-L50B0 were synergistic. The combination of the two peptides of enterocin L50 (L50A-L50B) showed partial synergy. This combination has shown synergy in previous studies [29]. However, the methods used to interpret this can vary, and the FIC_INDEX_ for L50A-L50B was 0.56, which is very close to synergy. Given that these two peptides are very active at low concentrations and that they have a broad spectrum of activity, this combination is very promising for further applications. For the combinations of enterocin B, whose mechanism of action is still unknown, with other enterocins, the FIC_INDEX_ value showed a partial synergy. This can be explained by the idea that enterocin B does not bind to the ManPTS system and has a different mechanism of action, which needs to be further studied by other methods. The FIC_INDEX_ was very low for other synergistic combinations such as EntA-L50A and EntP-L50A. However, as the MIC values for enterocin P are high, the combination EntA-L50A may be a better candidate, as lower concentrations of enterocin A than enterocin P are required to achieve inhibitory activity. Comparing the combinations with nisin, even though it is very effective, the combination of different enterocins in synergistic relationships could be advantageous because the antimicrobial activity can be enhanced, and, by combining different mechanisms, the spectrum of activity can also be broadened.

In summary, the enterocins produced by chemical synthesis in this study are active against, among others, the poultry pathogens *C. perfringens*, *L. monocytogenes*, and *E. cecorum*. Their activity depends on their mode of action, and enterocins using the ManPTS system as a receptor showed a similar spectrum of activity, while enterocin L50, which has a different mode of action, showed a broader spectrum with inhibitory activity, even against Gram-negative *P. aeruginosa* and *C. coli*. Combining enterocins with different modes of action resulted in increased antimicrobial activity against *C. perfringens* as they appeared to be synergistic or at least partially synergistic.

## 4. Materials and Methods

### 4.1. Strain Collection, Maintenance, and Propagation

A collection of 20 previously characterized *C. perfringens* strains recovered from broiler chicken flocks affected by necrotic enteritis and kindly provided by Dr. Marie-Lou Gaucher from the Research chair in meat safety, the Faculty of veterinary medicine, St-Hyacinthe, Quebec, Montreal were used in this study. Other isolates used in the study belong to strain type collections: *E. faecalis* ATCC 23212, *M. luteus* ATCC 10240, *S. aureus* ATCC 6538, *L. monocytogenes* ATCC 1911, *S. pyogenes* ATCC 19615, *S. enterica* ATCC 69162, *E. coli* ATCC 24922, *P. aeruginosa* ATCC 27855, and *C. coli* ATCC 33559. Additionally, *E. cecorum* CECO0009 from the Laval University collection, *S. suis* C2058, and methicillin-resistant *S. aureus* C411 from the La Rioja University collection were also used.

All isolates were preserved in 40% glycerol at −80 °C. Reinforced medium for clostridia (HiMedia, Kennett Square, PA, USA) was used for the propagation of *C. perfringens* isolates (incubation at 37 °C, 24 h, under strict anaerobic conditions). Brain Heart Infusion (Becton, Dickinson and Company, Helidelberg, Germany) was used for the propagation of non-clostridial aerobic isolates (incubation at 37 °C, 24 h, under aerobic conditions) and BD BBL supplemented with 5% blood (Becton, Dickinson and Company, Helidelberg, Germany) for *C. coli* (incubation at 42 °C, under microaerophilic conditions).

### 4.2. Genome Analysis of C. perfringens Isolates

Whole genome sequencing of the *C. perfringens* collection was performed previously [3] using the Illumina technique at the Hospital Center of University Laval (CHUL), Quebec, Canada. Some of the sequences were further analyzed in this study. Briefly, raw sequencing data were processed using fastp 0.20.0 for trimming and quality control of trimmed reads [42]. De novo assembly, without alignment to a reference genome, was performed using SPAdes 5.0.2 [43], with QUAST 1.14.6 used to check the quality of the assembly [44]. Prokka 1.14.6 [45] was used for gene prediction and annotation, using Prodigal for coding sequence prediction [46]. 

Pairwise alignments and visualization of the products of the genes encoding the Man-PTS subunit IID (*manZ_1*, *manZ_2*, and *manZ_3*) from the selected isolates were performed with the program Jalview 2.11.2.5 [47] in order to detect mutations and explain differences in enterocin susceptibility between strains. The isolates *C. perfringens* MLG 0418, 2919, and 7307 (which previously showed unique characteristics [3]) were chosen for analysis of the Man-PTS receptor. Sequences from *C. perfringens* ATCC 13124 were added as a reference.

### 4.3. Production of Enterocins

On the basis of their different mechanisms of action, five enterocins were selected for this study, and their amino acid sequences are shown in Table 4.

All reagents and solvents were purchased from commercial suppliers and used without additional purification. Fmoc-protected amino acids, 2-chlorotrityl chloride resin, and DIC were purchased from Matrix Innovations (Québec, QC, Canada), and the Oxyma Pure was acquired from CEM (Matthews, NC, USA). Other reagents and solvents were purchased from Sigma-Aldrich (St-Louis, MO, USA) or Fisher Scientific (Hampton, NH, USA).

Chemical synthesis of the six enterocins (enterocin L50A, L50B, A, B, P, and SEK4) was performed on a microwave-assisted peptide synthesizer (CEM Liberty Blue 2.0, Matthews, NC, USA). The peptides were prepared by standard solid-phase peptide synthesis (SPPS) on a 0.05 mmol scale using the Fmoc/tBu strategy on a preloaded 2-CTC polystyrene resin (typically 0.3 mmol/g). Briefly, the Fmoc protecting group was removed from the resin by treatment with a solution of 10% piperidine in DMF (*v*/*v*) for 5 min at 60 °C, and amino acid couplings were performed with Fmoc-Xaa-OH (5 equiv), Oxyma pure (5 equiv), DIEA (0.1 equiv), and DIC (10 equiv) in DMF for 20 min at 50 °C. After the synthesis, the resin was washed successively with DMF (5 × 5 mL) and CH_2_Cl_2_ (5 × 5 mL).

The peptides were cleaved from the resin by treatment with 10 mL of a solution of 20% HFIP in CH_2_Cl_2_ (2 × 20 min), and the amino acid side chains were deprotected by treating with 10 mL of a deprotection cocktail containing TFA/TIS/H_2_O/Phenol/DODT (90:2.5:2.5:2.5:2.5) for 3 h. The resulting peptide was precipitated in cold diethyl ether, and the solid was washed twice with diethyl ether before drying under a vacuum overnight.

The peptides were purified by semi-preparative RP-HPLC with a Shimadzu Prominence system on a Phenomenex Kinetex EVO C18 column (250 × 21.2 mm, 300 Å, 5 μm) using H_2_O (0.1% TFA) (A) and CH_3_CN (0.1% TFA) (B), with a linear gradient of 10–50% for 20 min at a rate of 12 mL/min and detection at 220 and 254 nm. The collected fractions were lyophilized to afford the desired peptide as a white powder. Peptide purity and composition were confirmed by HPLC and mass spectrometry on a Shimadzu Prominence LCMS-2020 system equipped with an electrospray ionization (ESI) probe using a Phenomenex Kinetex EVO C18 column (100 mm × 4.6 mm, 100 Å, 2.6 μm) with a 10.5 min gradient from water (0.1% HCOOH) and CH_3_CN (0.1%HCCOH) (10 to 100% CH_3_CN) and detection at 220 and 254 nm. 

The peptides, obtained as white powder, were stored at −20 °C. For the remainder of the assays, they were dissolved in distilled water.

### 4.4. Antimicrobial Activity Assay

The antimicrobial activity of the peptides was first studied by agar well diffusion against the strain collection. After, the minimal inhibitory concentration (MIC) of the strains was calculated for those enterocins showing antimicrobial activity against indicator strains, as in [48].

For agar well diffusion, 25 μL of a bacterial suspension 0.5 McFarland of each indicator strain was diluted in 25 mL of Mueller–Hinton soft agar (Oxoid) and placed in a petri dish. Once dried, wells were formed by using a 10 mL pipette. Later, 80 μL of each enterocin at a concentration of 200 μg/mL dissolved in distilled water was placed in each well. Nisin, at a concentration of 100 μg/mL was added as positive control. Incubation was performed at 37 °C during 24 h. For the *C. perfringens* and *C. coli* isolates, instead, brucella soft agar (HiMedia) media was used, and the incubation was under strict anaerobic or microaerophilic conditions, respectively.

A microtitration assay was performed to determine the MIC of the enterocins against the collection of indicator bacteria. Mueller–Hinton broth (Oxoid) was used as the growth medium for the aerobic isolates. For clostridial species and *C. coli*, brucella media for anaerobes (HiMedia) was used. In a 96-well plaque, 175 µL of the culture medium was added to the wells of column 1 (=negative control) and 125 µL to the wells of columns 2–12. Then 125 µL of each enterocin (stock concentration of 200 μg/mL, dissolved in distilled water) and nisin as a positive control (stock concentration of 100 μg/mL) were added to the wells in column 3 and mixed by pipetting up and down 10 times. A total of 125 µL from column 3 was removed and placed in column 4. All bacteriocins were dissolved in distilled water. The process was repeated until column 12 was reached. After mixing, 125 µL from column 12 was discarded. Later, 50 μL of the indicator strain suspension was inoculated in all the wells, except for column 1, to achieve a final bacterial concentration ≈ 10^5^ CFU/well. The microplate was incubated for 24 h at 37 °C in aerobic conditions for all isolates of the collection, except for clostridial species and *C. coli*, which were incubated under strict anaerobic and microaerophilic conditions, respectively. After incubation, the number of wells showing inhibition was recorded to calculate the MICs, with the MIC value being the concentration of enterocin (in µg/mL) that produced complete growth inhibition of the bacteria tested.

### 4.5. Checkerboard/FIC Assay

The activity of the different combined enterocins was evaluated by calculating the FIC_INDEX_ of eight different combinations, using the microdilution checkerboard method, as previously indicated [49]. The FIC index was calculated as follows:

$${FIC}_{INDEX}={FIC}_{A}+{FIC}_{B},$$

where ${FIC}_{A}={CMI}_{SYNERGY}/{CMI}_{A}$ ; ${FIC}_{B}={CMI}_{SYNERGY}/{CMI}_{B}$ .

The effect of the different combinations was interpreted as follows: FIC ≤ 0.5 for a synergetic effect, 0.5 < FIC ≤ 0.75 for partial synergy, 0.75 < FIC < 1 for additivity, 1 ≤ FIC ≤ 4 for neutrality, and FIC > 4 for antagonism.

The enterocin combinations were selected according to their modes of action: L50A-L50B, EntA-L50A, EntA-L50B, EntA-EntB, EntB-L50A, EntB-L50B, EntP-L50A, and EntP-L50B. The strain *C. perfringens* MLG3111 was selected as an indicator strain for this assay due to its high susceptibility to the enterocins tested.

## 5. Conclusions

Our study reinforces the idea of using enterocins as a promising alternative to antibiotics in the poultry sector, since they exhibit antimicrobial activity against relevant and problematic bacterial pathogens and can be easily produced by chemical synthesis. Moreover, our study also demonstrated that combinations of enterocins based on their mode of action can significantly enhance antimicrobial activity and efficacy. 

## Figures and Tables

**Table 1:** The MIC (μg/mL) of the enterocins against the collection of *C. perfringens* isolates ^a^.

*C. perfringens* Isolate	L50A	L50B	EntA	EntB	EntP	EntSEK4	Nisin
MLG0418	6.25	12.5	25	50	50	- ^b^	1.56
MLG0618	6.25	12.5	>100	100	>100	>100	0.39
MLG0712	1.56	25	>100	100	>100	-	0.39
MLG1108	3.12	25	>100	25	>100	-	0.78
MLG1619	12.5	50	>100	100	>100	-	0.19
MLG1819	6.25	12.5	>100	25	50	-	0.39
MLG2203	12.5	25	>100	50	>100	-	<0.09
MLG2314	3.12	12.5	>100	50	100	-	1.56
MLG2919	6.25	12.5	>100	>100	>100	-	1.56
MLG3111	3.12	12.5	25	50	50	-	0.39
MLG3406	6.25	12.5	>100	50	>100	-	3.12
MLG4201	6.25	25	50	100	100	-	0.78
MLG4206	6.25	12.5	50	50	>100	>100	1.56
MLG5719	6.25	25	>100	>100	>100	>100	1.56
MLG5806	6.25	25	>100	100	>100	>100	1.56
MLG6907	12.5	50	>100	>100	>100	-	1.56
MLG7009	6.25	25	>100	50	>100	-	1.56
MLG7307	1.56	6.25	3.12	6.25	1.56	-	0.78
MLG7309	6.25	50	>100	100	100	-	0.78
MLG7814	12.5	50	>100	>100	>100	>100	3.12

^a^ Data represent technical duplicate values. ^b^ Not active.

**Table 2:** The MIC (in μg/mL) of the enterocins against different pathogens ^a^.

Pathogens	L50A	L50B	EntA	EntB	EntP	EntSEK4
*Listeria monocytogenes* ATCC 1911	<0.19	<0.19	<0.19	3.12	<0.19	1.56
*Enterococcus faecalis* ATCC 29212	3.12	6.25	1.56	1.56	- ^b^	-
*Enterococcus cecorum* CECO 0009	1.56	1.56	-	-	-	-
*Streptococcus suis* C2058	1.56	1.56	50	1.56	-	-
*Streptococcus pyogenes* ATCC 19615	0.78	0.78	-	<0.19	-	-
*Micrococcus luteus* ATCC10240	1.56	3.12	-	-	-	-
*Staphylococcus aureus* ATCC 6538	6.25	6.25	-	-	-	-
*Staphylococcus aureus* C411 ^c^	12.5	25	-	-	-	-
*Pseudomonas aeruginosa* ATCC 27855	12.5	25	-	-	-	-
*Campylobacter coli* ATCC 33559	25	50	-	-	-	-

^a^ Data represent technical duplicate values. ^b^ No antimicrobial activity was detected. ^c^ Methicillin-resistant *S. aureus.*

**Table 3:** The FIC values of different combinations of enterocins against *C. perfringens* MLG3111.

Combination		FIC_INDEX_	Effect
Compound A	Compound B		
L50A	L50B	0.56	Partial synergy
EntA	L50A	0.37	Synergy
EntA	L50B	0.5	Synergy
EntA	EntB	0.56	Partial synergy
EntB	L50A	0.56	Partial synergy
EntB	L50B	0.62	Partial synergy
EntP	L50A	0.05	Synergy
EntP	L50B	0.15	Synergy

**Table 4:** Amino acid sequences of the enterocins synthesized in the study.

Enterocin	Class	Length	Amino Acid Sequence
L50A	IId	44 AA	MGAIAKLVAKFGWPIVKKYYKQIMQFIGEGWAINKIIIEWIKKHI
L50B	IId	43 AA	MGAIAKLVTKEGWPLIKKFYKQIMQFIGQGWTIFQIEKWLKRH
EntA	IIa	47 AA	TTHSGKYYGNGVYCTKNKCTVDWAKATTCIAGMSIGGFLGGAIPGKC
EntB	II	53 AA	ENDHRMPNELNRPNNLSKGGAKCGAAIAGGLFGIPKGPLAWAAGLANVYSKCN
EntP	IIa	44 AA	ATRSYGNGVYCNNSKCWVNWGEAKENIAGIVISGWASGLAGMGH
SEK4	IIa	43 AA	ATYYGNGVYCNKOKCWVDWSRARSEIIDRGVKAYVNGFTKVLG

## Supplementary Materials

The following supporting information can be downloaded at: https://www.mdpi.com/1422-0067/25/3/1597/s1.
