# Supplementary material for: Antimicrobial Activity of Synthetic Enterocins A, B, P, SEK4, and L50, Alone and in Combinations, against Clostridium perfringens
Source: Int J Mol Sci. 2024 Jan 27;25(3):1597. doi: 10.3390/ijms25031597 (PMC10855908; doi:10.3390/ijms25031597)
Supplement: Supplementary file 1 [file ijms-25-01597-s001.zip › a-Supplementary Figure S1.pdf]

Supplementary Figure S1. HPLC profiles (λ = 220 nm) and ESI-MS spectra of synthesized enterocins.

Enterocin A

H_2N-TTHSGKYYGNGVYCTKNKCTVDWAKATTCIAGMSIGGFLGGAIPGKC-COOH

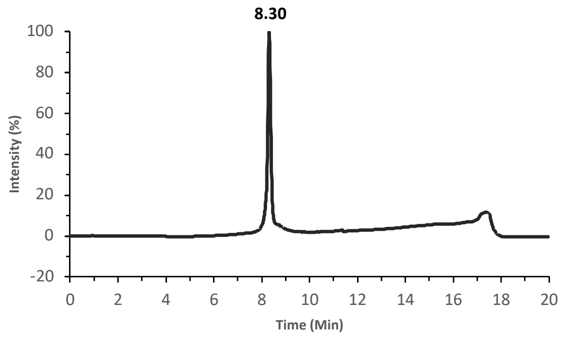

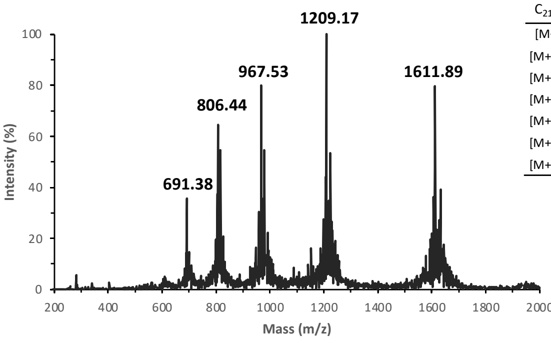

Calculated for $C_{211}H_{330}N_{57}O_{63}S_5$	
$[M+H]^+$	4833.64
$[M+2H]^{2+}$	2417.32
$[M+3H]^{3+}$	1611.65
$[M+4H]^{4+}$	1209.00
$[M+5H]^{5+}$	967.35
$[M+6H]^{6+}$	806.40
$[M+7H]^{7+}$	691.45

Enterocin B

H_2N-ENDHRMPNELNRPNNLSKGGAKCGAAIAGGLFGIPKGPLAWAAGLANVYSKCN-COOH

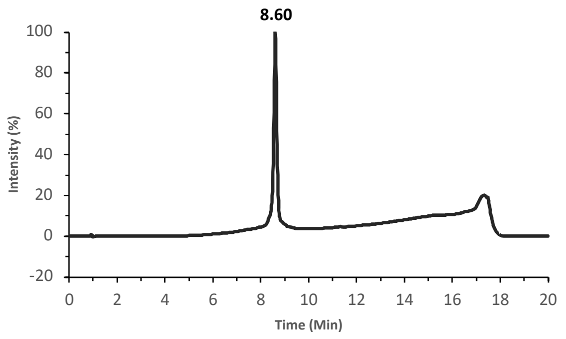

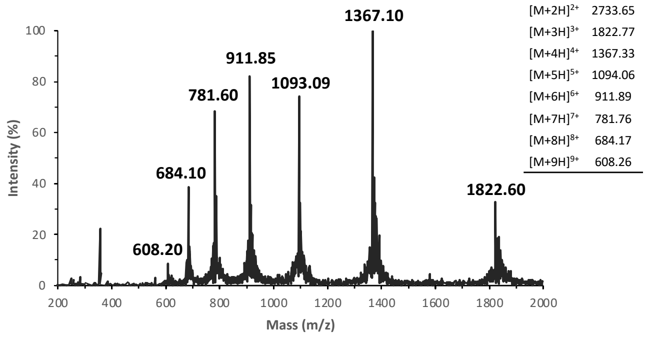

Calculated for $C_{237}H_{378}N_{73}O_{70}S_3$	
$[M+H]^+$	5466.29
$[M+2H]^{2+}$	2733.65
$[M+3H]^{3+}$	1822.77
$[M+4H]^{4+}$	1367.33
$[M+5H]^{5+}$	1094.06
$[M+6H]^{6+}$	911.89
$[M+7H]^{7+}$	781.76
$[M+8H]^{8+}$	684.17
$[M+9H]^{9+}$	608.26

Enterocin P

H_2N-ATRSYGNGVYCNNSKCWVNWGEAKENIAGIVISGWASGLAGMGH-COOH

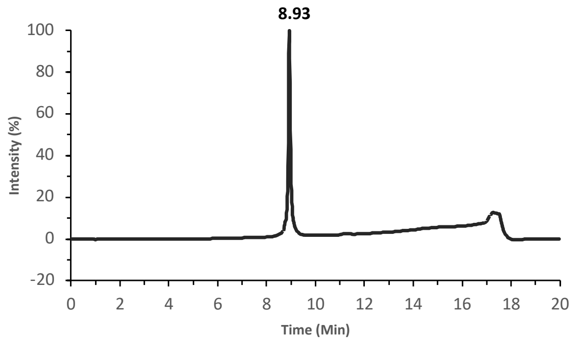

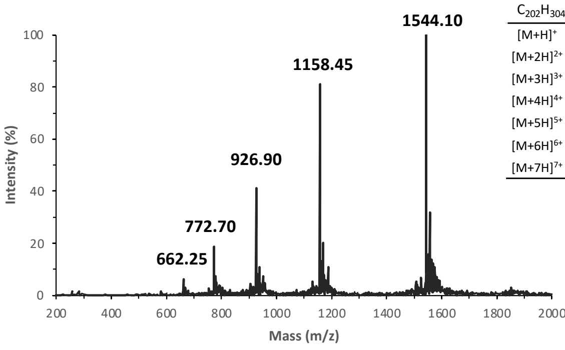

Calculated for $C_{202}H_{304}N_{59}O_{61}S_3$	
$[M+H]^+$	4631.22
$[M+2H]^{2+}$	2316.11
$[M+3H]^{3+}$	1544.41
$[M+4H]^{4+}$	1158.56
$[M+5H]^{5+}$	927.05
$[M+6H]^{6+}$	772.71
$[M+7H]^{7+}$	662.47

Supplementary Figure S1. HPLC profiles (λ = 220 nm) and ESI-MS spectra of synthesized enterocins. (Continued)

Enterocin SEK4

H2N-ATYYGNGVYCNKQKCWVDWSRARSEIIDRGVKAYVNGFTKVLG-COOH

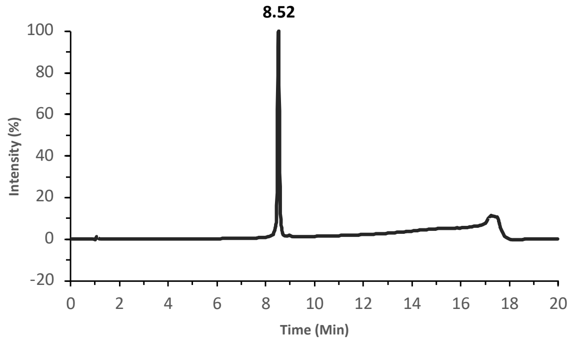

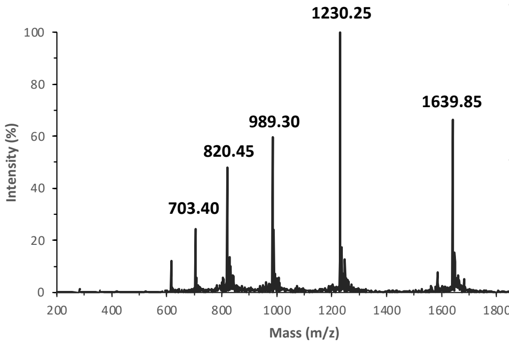

Calculated for $C_{221}H_{337}N_{62}O_{62}S_2$	
$[M+H]^{+}$	4918.65
$[M+2H]^{2+}$	2459.83
$[M+3H]^{3+}$	1640.22
$[M+4H]^{4+}$	1230.42
$[M+5H]^{5+}$	984.54
$[M+6H]^{6+}$	820.61
$[M+7H]^{7+}$	703.53

Enterocin L50A

H2N-MGAIAKLVAKFGWPIVKKYYKQIMQFIGEGWAINKIIEWIKKHI-COOH

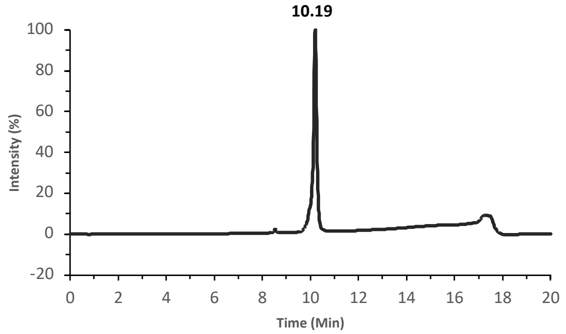

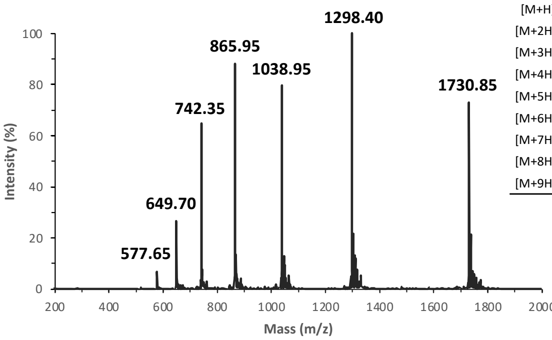

Calculated for $C_{252}H_{393}N_{60}O_{54}S_2$	
$[M+H]^{+}$	5191.43
$[M+2H]^{2+}$	2596.22
$[M+3H]^{3+}$	1731.15
$[M+4H]^{4+}$	1298.61
$[M+5H]^{5+}$	1039.09
$[M+6H]^{6+}$	866.08
$[M+7H]^{7+}$	742.50
$[M+8H]^{8+}$	649.81
$[M+9H]^{9+}$	577.72

Enterocin L50B

H2N-MGAIAKLVTKFGWPLIKKFYKQIMQFIGQGWTIDQIEKWLKRH-COOH

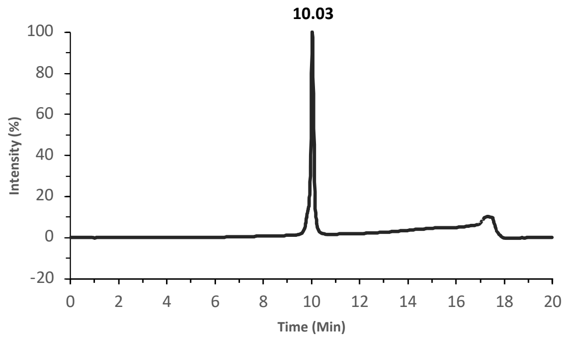

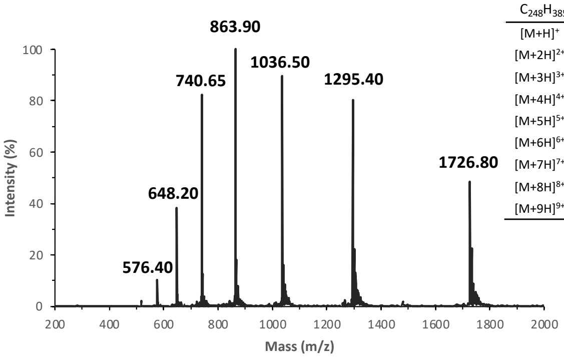

Calculated for $C_{248}H_{385}N_{62}O_{55}S_2$	
$[M+H]^{+}$	5179.34
$[M+2H]^{2+}$	2590.17
$[M+3H]^{3+}$	1727.12
$[M+4H]^{4+}$	1295.59
$[M+5H]^{5+}$	1036.67
$[M+6H]^{6+}$	864.06
$[M+7H]^{7+}$	740.77
$[M+8H]^{8+}$	648.30
$[M+9H]^{9+}$	576.38
