# Supplementary material for: Antimicrobial Activity of Synthetic Enterocins A, B, P, SEK4, and L50, Alone and in Combinations, against Clostridium perfringens
Source: Int J Mol Sci. 2024 Jan 27;25(3):1597. doi: 10.3390/ijms25031597 (PMC10855908; doi:10.3390/ijms25031597)
Supplement: Supplementary file 1 [file ijms-25-01597-s001.zip › d-Supplementary Figure S4.pdf]

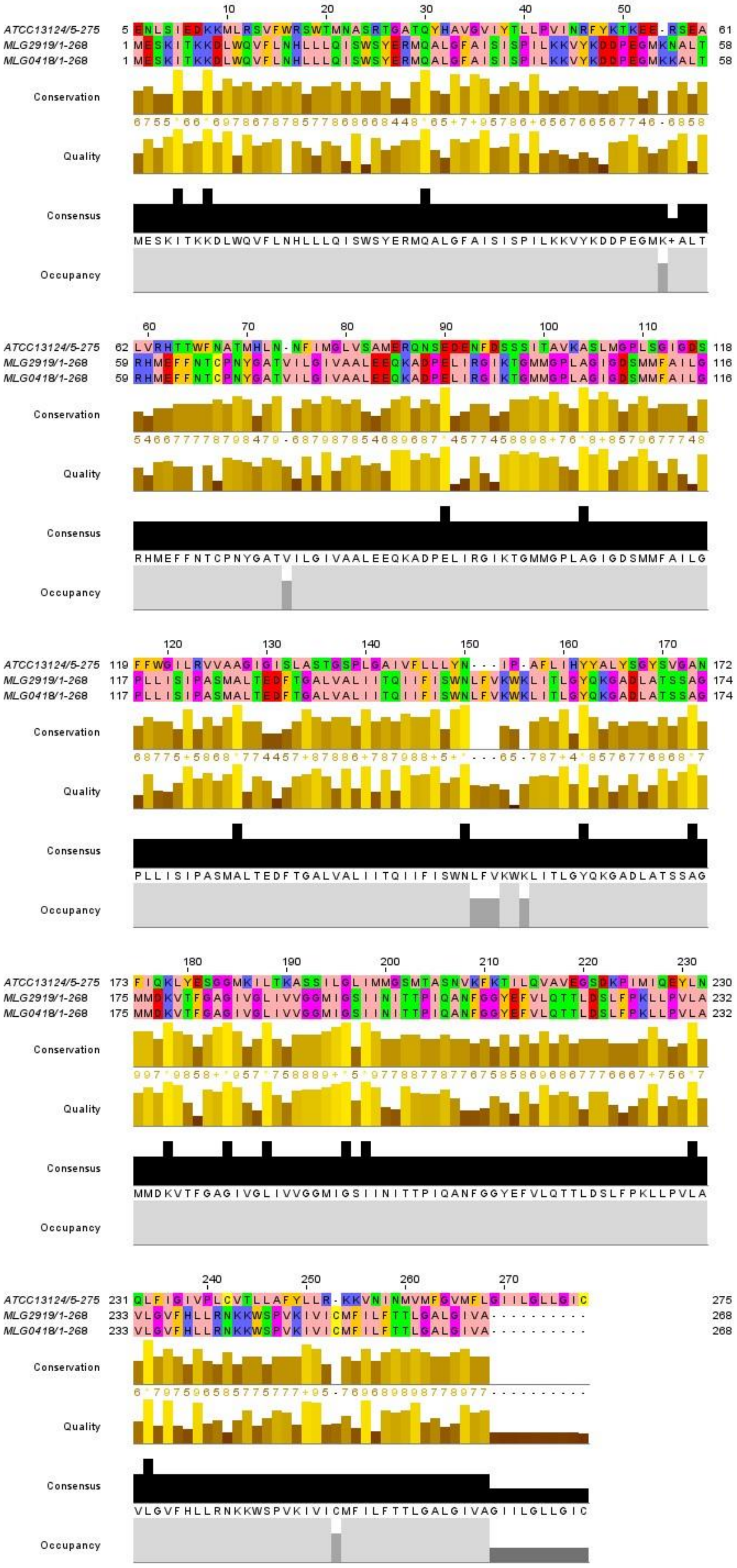

**Figure S4**. Pairwise alignments of the product of the gene *manZ_3* from three *C. perfringens* isolates from the collection and the *C. perfringens* ATTC 13124.
