# Supplementary material for: Antimicrobial Activity of Synthetic Enterocins A, B, P, SEK4, and L50, Alone and in Combinations, against Clostridium perfringens
Source: Int J Mol Sci. 2024 Jan 27;25(3):1597. doi: 10.3390/ijms25031597 (PMC10855908; doi:10.3390/ijms25031597)
Supplement: Supplementary file 1 [file ijms-25-01597-s001.zip › e-Supplementary Table S1.pdf]

Table S1. Inhibition halos (in mm) of the different enterocins against the *C. perfringens* collection. Nisin was added as control.

C. perfringens isolate	Enterocin A	Enterocin B	Enterocin P	Enterocin SEK4	Enterocin L50A	Enterocin L50B	Nisin
MLG0418	18	16	15	-[a]	16	14	23
MLG0618	23	20	22	11	21	19	14
MLG0712	21	12	19	-	16	14	17
MLG1108	22	15	19	-	16	15	16
MLG1619	18	12	18	-	17	16	20
MLG1819	21	12	18	-	16	15	19
MLG2203	21	16	20	-	15	14	24
MLG2314	21	16	17	-	16	14	20
MLG2919	20	10	18	-	16	13	21
MLG3111	18	15	19	-	15	14	27
MLG3406	20	12	20	-	16	15	19
MLG4201	21	11	19	-	16	15	18
MLG4206	22	12	19	8	16	14	25
MLG5719	20	11	17	9	15	14	19
MLG5806	19	9	17	8	14	14	19
MLG6907	19	9	17	-	16	13	23
MLG7009	21	12	19	-	16	14	20
MLG7307	22	11	22	-	17	15	20
MLG7309	21	12	18	-	15	14	17
MLG7814	19	11	19	11	15	13	23

a: Not active
