# Supplementary material for: Antimicrobial Activity of Synthetic Enterocins A, B, P, SEK4, and L50, Alone and in Combinations, against Clostridium perfringens
Source: Int J Mol Sci. 2024 Jan 27;25(3):1597. doi: 10.3390/ijms25031597 (PMC10855908; doi:10.3390/ijms25031597)
Supplement: Supplementary file 1 [file ijms-25-01597-s001.zip › f-Supplementary Table S2.pdf]

Supplementary Table S2. Inhibition halos (in mm) of the enterocins against relevant bacteria.

Pathogens	Enterocins					
	L50A	L50B	EntA	EntB	EntP	EntSEK4
L. monocytogenes ATCC1911	15	17	25	16	21	13
E. faecalis ATCC29212	15	14	18	16	20	12
E. cecorum C0009	28	26	-[a]	17	-	16
S. suis C2058	15	14	12	17	13	-
S. pyogenes ATCC19615	26	26	-	11	-	-
M. luteus ATCC10240	20	16	-	-	-	-
S. aureus ATCC6538	15	15	-	-	-	-
S. aureus C411	13	11	-	-	-	-
P. aeruginosa ATCC27855	14	13	-	-	-	-
C. coli ATCC33559	10	10	-	-	-	-

a: Not active

Note: No activity was detected against *E. coli* ATCC 24922 and *S. enterica* ATCC 69162
